# Low Openness on the Revised NEO Personality Inventory as a Risk Factor for Treatment-Resistant Depression

**DOI:** 10.1371/journal.pone.0071964

**Published:** 2013-09-03

**Authors:** Michio Takahashi, Yukihiko Shirayama, Katsumasa Muneoka, Masatoshi Suzuki, Koichi Sato, Kenji Hashimoto

**Affiliations:** 1 Department of Psychiatry, Teikyo University Chiba Medical Center, Ichihara, Japan; 2 Division of Clinical Neuroscience, Chiba University Center for Forensic Mental Health, Chiba, Japan; Rikagaku Kenkyūsho Brain Science Institute, Japan

## Abstract

**Background:**

Recently, we reported that low reward dependence, and to a lesser extent, low cooperativeness in the Temperature and Character Inventory (TCI) may be risk factors for treatment-resistant depression. Here, we analyzed additional psychological traits in these patients.

**Methods:**

We administered Costa and McCrae's five-factor model personality inventory, NEO Personality Inventory-Revised (NEO-PI-R), to antidepressant-treatment resistant depressed patients (n = 35), remitted depressed patients (n = 27), and healthy controls (n = 66). We also evaluated the relationships between scores on NEO and TCI, using the same cohort of patients with treatment-resistant depression, as our previous study.

**Results:**

Patients with treatment-resistant depression showed high scores for neuroticism, low scores for extraversion, openness and conscientiousness, without changes in agreeableness, on the NEO. However, patients in remitted depression showed no significant scores on NEO. Patients with treatment-resistant depression and low openness on NEO showed positive relationships with reward dependence and cooperativeness on the TCI.

**Conclusions:**

Many studies have reported that depressed patients show high neuroticism, low extraversion and low conscientiousness on the NEO. Our study highlights low openness on the NEO, as a risk mediator in treatment-resistant depression. This newly identified trait should be included as a risk factor in treatment-resistant depression.

## Introduction

It is well documented that 60 to 70 percent of depressed patients respond to first line antidepressant treatment at maximum dose, for at least two months. Between 80 and 90 percent of these patients respond to first or second choice prescribed antidepressant medication. The remaining 10 to 15 percent of patients who do not respond to therapy are deemed to have treatment-resistant depression [1], [2]. Response is defined as a reduction in depressive symptoms to less than 50 percent, but not necessarily recovery. Remission is described as a full recovery. We recently reported that low reward dependence and to a lesser extent, low cooperativeness in the Temperature and Character Inventory (TCI) [3] may be risk factors for treatment-resistant depression [4]. Furthermore, patients with remitted depression show high scores for harm avoidance, relative to healthy controls [4]. It is likely that additional psychological factors associated with depression are yet to be identified from this group of patients.

Another personality inventory, the NEO Personality Inventory-Revised (NEO-PI-R) is also in common use [5]. This five-factor model of personality structures personality in terms of five traits: neuroticism, extraversion, openness, agreeableness, and conscientiousness. Numerous studies have reported that depressed patients show high scores for neuroticism and low scores for extraversion and conscientiousness using the NEO [6]–[13]. The severity of depression correlates positively with neuroticism and negatively with extraversion [9], [10], [14], [15]. The personality traits of neuroticism and extraversion are associated with negative and positive emotional experiences, respectively [16]. Furthermore, neuroticism scores differed between the depressed and post antidepressant treatment states [9], [15], [17], [18].

Chronically depressed patients also reported higher levels of neuroticism and lower levels of extraversion, agreeableness, and conscientiousness, compared with those suffering acute forms of the disease [19]. Treatment-resistant depression patients had significantly higher neuroticism and lower extraversion scores [14]. Interestingly, the duration of depressive episodes significantly correlates with high levels of premorbid neuroticism [20]. Scores of neuroticism increased, while scores of extraversion and conscientiousness decreased with the occurrence of depression, but the scores for conscientiousness changed very little on recovery from depressive disorders [21]. At times, individuals with remitted depression showed significantly more neuroticism than healthy controls [22]. It is well known that residual symptoms during remission have a strong prognostic value [23]. These results indicate that some psychological features are resistant to treatment and persistent in patients with remitted depression.

The purpose of this study was to investigate in more depth, the presence of personality biases in patients with treatment-resistant depression, using the NEO-PI-R [5]. Additionally, we evaluated the relationships between scores obtained using NEO in this study, and those obtained using TCI in our previous study [4], using the same cohort of treatment-resistant depression patients.

## Methods

### Ethics statement

The study was approved by the ethics committee of Teikyo University Chiba Medical Center (study number 09-30) and performed in accordance with the Declaration of Helsinki. Written informed consent was obtained from all participants, after procedures had been fully explained.

### Subjects

Sixty six healthy subjects, 27 depressed patients in remission, and 35 antidepressant treatment-resistant depressed patients were enrolled on this study (**Table 1**). The treatment-resistant depressed patients were the same sample used in our previous study [4]. All patients met the DSM-IV criteria for major depressive disorder (MDD) (first episode) [24]. Patients were recruited from the outpatient clinics of Teikyo University Chiba Medical Center. All patients were physically healthy and free of alcohol or drug abuse. Inclusion criteria required symptoms of moderate depression, after treatment with at least two antidepressants, for 8 weeks [2]. Scores for patients were 14 or more on the 17-item Hamilton Rating Scale for Depression (HAM-D), on which remission or recovery was scored at 7 or less [1]. Healthy control subjects with no past history of psychiatric disorders or drug dependence were recruited. Clinical information on all subjects is provided in **Table 1**. The duration of depressive states in patients with treatment-resistant depression was significantly longer than in those with remitted depression.

**Table 1:** Demographic information of subjects.

	Healthy control(n = 66)	Remitted depression(n = 27)	Treatment-resistant depression(n = 35)	P values
Current age (years)	38.09±8.46 (23–61)	39.07±9.19 (22–56)	38.74±9.42 (22–53)	0.821
Sex (male/female)	56/10	18/9	24/11	0.073
Age onset (years)		36.07±9.27 (22–54)	35.94±8.93 (17–50)	0.955
Duration of depressive state (months)		19.44±15.67 (3–68)	36.46±21.32* (9–98)	0.002
Duration of treatment (months)		26.63±24.34 (6–54)	30.06±26.23 (4–97)	0.517
HAM-D		4.48±2.76 (3–7)	18.31±4.04 ** (14–28)	<0.001
Trial numbers of antidepressants		1.26±0.45 (1–2)	2.54±1.25 ** (2–9)	<0.001

Data are shown as mean ± SD.

Parenthesis is the range.

HAM-D: Hamilton Rating Scale for Depression.

* P<0.01,

** P<0.001 as compared to the remitted group (Student's t-test).

### Personality Scores and Psychological Tests

Personality was assessed using NEO PI-R. NEO PI-R utilized the five-factor model of personality: neuroticism, extraversion, openness, agreeableness, and conscientiousness [5]. Each domain scale is comprised of six item facets. The NEO-PI-R consists of 240 items answered on a five- point Likert scale, ranging from absolutely disagree to strongly agree. Raw scores were converted to T-scores for standardization. The mean and SD for each dimension are 50 and 10, respectively.

TCI Scores in patients with treatment-resistant depression were taken from our recently reported study [4]. In this study, we used TCI-125, a shortened version of the TCI [3], [25], [26]. Items are rated on a four-point scale. This test covers four dimensions of temperament: harm avoidance, novelty seeking, reward dependence, and persistence, and three dimensions of character: self-directedness, cooperativeness, and self-transcendence.

### Statistical Analysis

Data from five domains of the NEO were first analyzed using multiple analysis of variance (MANOVA), to check for the simultaneous existence of significant differences. Statistical differences among the three groups were determined by one-way factorial analysis of variance (ANOVA), followed by multiple comparison testing (Scheffe's test). Chi-square test was used for categorical variables. Statistical evaluation between the two groups was performed using a two-tailed Student's t-test. Coefficients among scores for NEO and TCI were estimated by Pearson coefficient. Differences were considered to be significant when p values were less than 0.01.

## Results

### Psychological Features assessed by NEO

**Figure 1:**
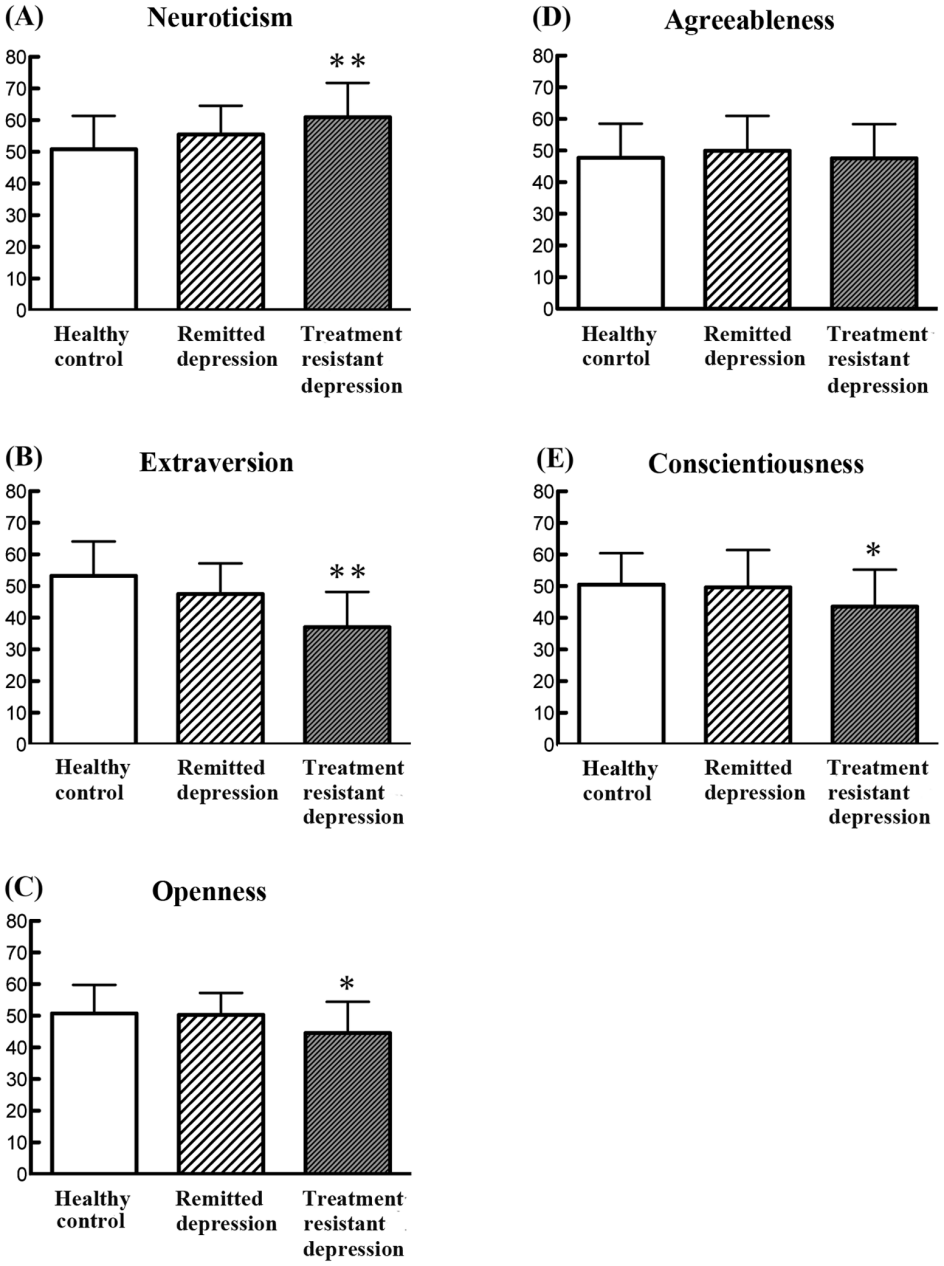
Analysis of variance shows a significant difference between three groups for (A) Neuroticism (F (2,125) = 11.10; P<0.001), (B) Extraversion (F (2,125) = 26.42; P<0.001), (C) Openness (F (2,125) = 5.93; P = 0.004), and (E) Conscientiousness (F (2,125) = 4.88; P = 0.009). In (D) Agreeableness, there is not a significant difference between three groups (F (2,125) = 0.49; P = 0.616). *P<0.01, **P<0.001 compared to control (ANOVA followed by Scheffe test).

MANOVA indicated a significant group effect (F = 5.777, P<0.0001). Subsequent one-way ANOVA demonstrated that patients with treatment-resistant depression showed significantly high scores for neuroticism and lower scores for extraversion, openness and conscientiousness on the NEO, compared with healthy controls or patients with remitted depression (**Figure 1**). Patients in remission showed no significant differences in NEO scores, compared to healthy controls (**Figure 1**).

The subscales of each domain on the NEO are shown in **Table 2**. Patients with treatment-resistant depression showed significantly higher scores for anxiety, depression, self-consciousness and vulnerability in the neuroticism subset. They also showed low scores for warmth, gregariousness, assertiveness, activity, excitement-seeking, and positive emotion in the extraversion subset, feelings and actions in the openness subset, modesty in the agreeableness subset, and competent, achievement striving and self-discipline in the conscientiousness subset, compared with remitted depression and healthy control subjects (**Table 2**).

**Table 2:** Comparison of NEO subscales in subjects.

	Healthy control(n = 66)	Remitted depression(n = 27)	Treatment-resistant depression(n = 35)	F	P
**<Neuroticism>**	50.82±10.55	55.44±9.04	60.94±10.83**	11.10	<0.001
Anxiety	51.24±10.88	56.11±9.72	60.57±9.89**	9.50	<0.001
Angry Hostility	50.62±12.16	53.93±8.10	55.63±11.98	2.42	0.094
Depression	50.79±10.49	55.93±10.27	62.57±10.12**	14.96	<0.001
Self-consciousness	50.42±10.48	51.85±8.97	58.00±10.75*	6.38	0.002
Impulsiveness	51.88±10.16	53.07±7.34	51.03±12.34	0.30	0.741
Vulnerability	49.53±10.65	55.22±10.53	62.11±11.07**	15.86	<0.001
**<Extraversion>**	53.26±47.48	47.48±9.66	37.00±11.16**	26.42	<0.001
Warmth	52.79±11.75	49.00±8.79	41.09±12.84**	11.81	<0.001
Gregariousness	52.33±12.54	47.44±10.67	40.89±10.71**	11.04	<0.001
Assertiveness	50.62±11.16	48.30±11.18	41.43±10.34**	8.13	<0.001
Activity	52.00±10.79	50.59±9.07	40.46±11.65**,#	13.92	<0.001
Excitement-Seeking	54.86±11.65	46.00±10.41*	42.31±10.52**	16.38	<0.001
Positive Emotions	52.65±11.10	48.48±10.54	39.31±9.48**,#	18.24	<0.001
**<Openness>**	50.76±9.01	50.33±6.90	44.60±9.83*	5.93	0.004
Fantasy	48.71±8.46	50.00±8.55	48.09±10.36	0.35	0.706
Aesthetics	46.85±10.69	49.26±9.28	43.29±9.41	2.83	0.063
Feelings	52.82±9.71	52.15±9.38	46.27±9.34*	5.66	0.004
Actions	54.03±10.65	50.00±10.36	46.11±9.47*	6.95	0.001
Ideas	49.91±9.95	49.33±8.43	44.31±11.23	3.76	0.026
Values	53.26±8.03	54.15±9.21	51.26±10.21	0.91	0.405
**<Agreeableness>**	47.71±10.77	49.96±11.02	47.54±10.83	0.48	0.973
Trust	51.29±11.70	47.63±10.23	44.14±12.15	4.50	0.013
Straightforwardness	46.58±10.31	50.41±9.10	49.20±6.77	2.00	0.140
Altruism	48.77±10.57	48.82±9.28	43.74±10.25	3.09	0.049
Compliance	49.58±10.51	50.74±10.65	49.17±11.89	0.17	0.134
Modesty	46.55±10.44	51.63±12.45	56.23±10.70**	9.20	<0.001
Tender-Mindedness	48.05±10.52	49.44±11.69	48.03±12.03	0.17	0.846
**<Conscientiousness>**	50.47±9.94	49.63±11.82	43.54±11.82*	4.88	0.009
Competent	51.23±10.78	49.15±11.04	41.94±12.65**	7.32	<0.001
Order	51.71±9.45	51.92±10.07	50.91±12.14	0.09	0.913
Dutifulness	47.96±9.28	49.07±9.20	43.86±9.29	3.03	0.052
Achievement Striving	52.96±10.87	48.48±10.69	40.06±12.73**	14.71	<0.001
Self-Discipline	50.82±10.21	50.07±13.83	41.37±10.99**	8.58	<0.001
Deliberation	48.15±9.94	48.89±9.77	52.23±10.98	1.87	0.158

Data are shown as mean ± SD.

* P<0.01,

** P<0.001 compared to control (ANOVA followed by Scheffe test).

# P<0.01,

## P<0.01compared to remitted depression (ANOVA followed by Scheffe test).

Neuroticism correlated significantly with HAM-D scores in all MDD patients including both remitted and treatment-resistant groups (neuroticism, r = 0.341, p<0.01; extraversion, r = −0.497, p<0.001). In contrast, there was no correlation between NEO scores and the severity of depression in patients with treatment-resistant depression (data not shown).

A significant negative correlation between neuroticism and extraversion was seen in healthy controls and remitted depression patients, but not in treatment-resistant depression patients (**Table 3**). Significant positive correlation between extraversion and openness was seen in healthy controls, but not in the remitted depression and treatment-resistant depression groups (**Table 3**).

**Table 3:** Correlates of NEO factors.

Healthy control (n = 66)	N	E	O	A	Co
Neuroticisms (N)	–				
Extraversion (E)	−.395**	–			
Openness (O)	.001	.457**	–		
Agreeableness (A)	−.346*	.277	.265	–	
Conscientiousness (Co)	−.489**	.304	.125	.022	–

Remitted depression (n = 27)	N	E	O	A	Co
Neuroticisms (N)	–				
Extraversion (E)	−.610**	–			
Openness (O)	.122	.136	–		
Agreeableness (A)	.201		−.188	.291	–
Conscientiousness (Co)	−.543*	.088	−.126	−.246	–

Treatment-resistant depression (n = 35)	N	E	O	A	Co
Neuroticisms (N)	–				
Extraversion (E)	−.239	–			
Openness (O)	.026	.411	–		
Agreeableness (A)	−.469*	.142	.376	–	
Conscientiousness (Co)	−.670**	.369	−.037	.167	–

* P<0.01,

** P<0.001.

### Relationship between scores on the NEO and the TCI in Patients with Treatment-Resistant Depression

As shown in **Table 4**, there were significant, strong relationships between NEO and TCI factors, in the patients with treatment-resistant depression. Openness on NEO correlated positively with reward dependence and cooperativeness in TCI. Similarly, agreeableness on the NEO correlated positively with reward dependence and cooperativeness on TCI. Neuroticism on the NEO showed positive correlation with harm avoidance and negative correlation with self-directedness and cooperativeness on the TCI. Extraversion on the NEO correlated negatively with harm avoidance and positively with reward dependence and persistence on the TCI. Conscientiousness on the NEO showed negative correlation with harm avoidance and positive correlation with persistence and self-directedness on the TCI.

**Table 4:** Correlates of TCI variables in treatment-resistant depressive patients.

	<TCI>						
	Novelty seeking	Harm avoidance	Reward dependence	Persistence	Self-directedness	Cooperativeness	Self-transcendence
**<NEO>**							
Neuroticism	.343	.682**	−.123	−.344	−.699**	−.502*	−.091
Extraversion	.130	−.574**	.694**	.435*	.416	.406	.353
Openness	.079	−.215	.542**	.042	.057	.505*	.207
Agreeableness	−.260	−.408	.446*	.178	.338	.618**	.051
Conscientiousness	−.369	−.486*	.233	.598**	.563*	.277	.226

* P<0.01,

** P<0.001.

## Discussion

We found that patients with treatment-resistant depression showed significantly altered scores in neuroticism, extraversion, openness and conscientiousness, as measured by NEO. Previous studies using the NEO show that depressed patients scored highly for neuroticism, low extraversion and low conscientiousness [6], [8], [9], [11], [13], [15]. Of the six published studies using this scale in depression (**Table 5**), all found significant alterations in scores for extraversion and conscientiousness, and all but one found significant changes in scores for neuroticism, highlighting a common pattern in depression. Since treatment-resistant patients suffer from depressive symptoms, it is not surprising that non-responders showed the same pattern of high scores for harm neuroticism and low scores for extraversion and conscientiousness, as depressed patients. It is also highly likely that the remaining factor, low openness, could be specific to patients with treatment-resistant depression. Examining the finding for openness, the subscales scores altered are feelings and actions (**Table 2**). Thus, it is likely that altered feelings and actions could be specific to treatment-resistant patients. It should be noted that low openness was associated with high ratios of self-reported, to observer-rated mood symptoms [10]. Although only one of six published studies detected low openness in depressed patients (**Table 5**) [15], the subjects in Griens's study seemed to involve patients with chronic or repetitive episodes of depression, based on the recorded long mean duration of illness (over 6 years), the repeated depressive episodes, but without high neuroticism.

**Table 5:** The published data of NEO scores of depressed patients.

	N	E	O	A	C
Bagby et al, 1998	↑	↓	–	–	↓
Petersen et al, 2001	↑	↓	–	–	↓
Du et al, 2001	↑	↓	–	–	↓
Griens et al, 2002	–	↓	↓	–	↓
Chopra et al, 2005	↑	↓	–	–	↓
Rector et al, 2012	↑	↓	–	–	↓
This study (treatment-resistant)	↑	↓	↓	–	↓

N: Neuroticism, E: Extraversion, O: Openness, A: Agreeableness, C: Conscientiousness.

↑: Increase, ↓: Decrease, –: No change.

We also detected a significant negative correlation between neuroticism and extraversion, in the healthy control and remitted depression groups, but not in the treatment-resistant depression group (**Table 3**). This negative relationship was also detected in depressed patients, in a previous study [10]. The absence of a relationship between neuroticism and extraversion in treatment-resistant depression may indicate that these patients have lost an adaptive mechanism that still functions in healthy controls. We speculate that neuroticism and extraversion on the NEO are probably less dependent on each other than originally thought in treatment-resistant depression. Furthermore, it appears that neuroticism and extraversion act together with cooperativeness and reward dependence, respectively (Table 4), when assessing treatment-resistant depression using TCI, as our previous study reported that both reward dependence and cooperativeness may be risk factors for treatment-resistant depression [4].

Patients with treatment-resistant depression showed a negative relationship between neuroticism and agreeableness, which also was seen in healthy controls, but not in remitted depressed patients (**Table 3**) or in depressed patients examined in different study [10]. We put forward that there may be a new connection between neuroticism and agreeableness, rather than between neuroticism and extraversion, leading to psychosocial isolation. These connected characteristics may partially contribute to the psychological features of treatment-resistant depression. Future studies will be needed to elucidate the roles of extraversion and agreeableness in the depressive state.

Here, openness on the NEO showed a positive relationship with reward dependence and cooperativeness on the TCI, in the treatment-resistant depression group (**Table 4**). Again, it should be noted that low scores for reward dependence and cooperativeness on the TCI are characteristic features in patients with treatment-resistant depression [4]. A previous study showed that openness on the NEO has significant relationships with novelty seeking, harm avoidance and self-transcendence on the TCI, in healthy volunteers [27]. Therefore, the remaining relationships between openness on the NEO and reward dependence, and cooperativeness on the TCI indicate that these factors may act together in treatment-resistant depression. Agreeableness on the NEO also showed a similar pattern for reward dependence and cooperativeness on the TCI, and with openness on the NEO, in treatment-resistant depression, although agreeableness on the NEO did not reach statistically significant levels in this study. A recent study reported that agreeableness on the NEO did not show a significant relationship with reward dependence on the TCI in healthy controls [27]. Therefore, agreeableness, as well as openness might play a role in the pathology of treatment-resistant depression.

We also found significant relationships between neuroticism on the NEO and harm avoidance and self-directedness on the TCI in treatment-resistant depression. Additionally, we detected association between extraversion on the NEO, and harm avoidance and reward dependence on the TCI, and between conscientiousness on the NEO and harm avoidance, persistence and self-directedness on the TCI in the same group of patients (**Table 4**). These same patterns were also seen in the healthy controls of a previous study [27], indicating that these characteristics are common to both groups. It is likely that this pattern represents the norm and is therefore seen in patients and normal controls. By contrast, significant relationships between neuroticism on the NEO and cooperativeness on the TCI, and between extraversion on the NEO and persistence on the TCI, were seen only in treatment-resistant depression. In addition, these patterns were not seen in healthy volunteers of the previously mentioned study [27]. These newly detected relationships in treatment-resistant depression patients indicate that high neuroticism and low extraversion on the NEO interact with low cooperativeness and persistence on the TCI, respectively, in the pathology of treatment-resistant depression. However, it remains unknown whether personality bias occurs as a result of long illness or exists as a cause of treatment-resistance.

Finally, this study failed to show any significant factors in remitted depression patients, using the NEO (**Figure 1**). However, our previous study using the TCI revealed that remitted patients still showed high scores for harm avoidance on the TCI, compared with normal controls [4]. Another study using the Maudsley Personality Inventory, showed that personality traits do not change after a typical episode of major depression [28]. Future studies will be needed to examine the psychological factors which contribute to the relapse of depression.

We put forward that patients with treatment-resistant depression display lower levels of resilience, compared with healthy subjects and remitted depression patients. A previous study showed that resilient individuals exhibit lower levels of denial, avoidant coping, pessimism and behavioral disengagement [29]. Positive emotions, which are generally seldom seen in depression, promote adaptive coping, openness to social support and flexible thinking [30]. Negative, rather than positive, life events predict a longer time to remission of depression, however, personality traits do not influence the effect of life events on disease course indicators [31]. Social support and educational levels were associated with long-term outcome of treatment-resistant depression [32]. Furthermore, personality dysfunction was also associated with poor response to antidepressant treatment in major depression [33]. Future studies are required to aid identification of factors related to resilience in treatment-resistant depression.

In conclusion, patients with treatment-resistant depression demonstrated high scores for neuroticism, low scores for extraversion, openness and conscientiousness using the NEO. Previous studies report that depressed patients show high neuroticism, low extraversion and low conscientiousness on the NEO. This would strongly imply that the remaining factor, namely, low openness is a specific feature of treatment-resistant depression. Openness on the NEO has positive relationships with reward dependence and cooperativeness on the TCI, in treatment-resistant depression. Our results indicate that these three factors are important mediators in treatment-resistant depression.
